# Imaging Findings of Intracholecystic Tubular Neoplasm (ICTN) of the Gallbladder: A Four‐Case Series With Imaging–Pathological Correlation

**DOI:** 10.1155/rrp/3658453

**Published:** 2026-11-15

**Authors:** Iyo Tanimura, Yoshisuke Kadoya, Masahiro Furukawa, Ruriko Aki, Makiko Minami, Kazuyoshi Katayanagi, Miku Hashimoto, Atsuhiro Kawashima, Hirotaka Kitamura, Yoshinao Ohbatake, Ichiro Onishi, Wataru Koda

**Affiliations:** ^1^ Department of Diagnostic Radiology Ishikawa Prefectural Central Hospital Kanazawa Ishikawa Japan ishikawa.jp; ^2^ Department of Radiology Kanazawa Medical University Kahoku Ishikawa Japan kanazawa-med.ac.jp; ^3^ Department of Radiology Kanazawa University Kanazawa Ishikawa Japan kanazawa-u.ac.jp; ^4^ Department of Radiology National Hospital Organization Kanazawa Medical Center Kanazawa Ishikawa Japan; ^5^ Department of Diagnostic Pathology Ishikawa Prefectural Central Hospital Kanazawa Ishikawa Japan ishikawa.jp; ^6^ Department of Pathology National Hospital Organization Kanazawa Medical Center Kanazawa Ishikawa Japan; ^7^ Department of Gastroenterological Surgery Ishikawa Prefectural Central Hospital Kanazawa Ishikawa Japan ishikawa.jp; ^8^ Department of Surgery National Hospital Organization Kanazawa Medical Center Kanazawa Ishikawa Japan

**Keywords:** diffusion-weighted imaging, dynamic contrast enhancement, gallbladder neoplasm, intracholecystic tubular nonmucinous neoplasm, MRI, pathological correlation

## Abstract

**Objectives:**

Intracholecystic tubular neoplasm (ICTN) is a recently recognized preinvasive, high‐grade gallbladder neoplasm characterized by a pedunculated polypoid lesion composed of small tubules with minimal or no mucin production. It is rarely associated with invasive carcinoma, and its imaging features have not been well described. Here, we aimed to evaluate the imaging findings of ICTN and correlate them with histopathologic findings.

**Methods:**

We retrospectively reviewed four surgically resected, pathologically confirmed ICTN cases from two institutions (2021–2025). Preoperative ultrasonography, computed tomography, and MRI were independently reviewed by two radiologists for tumor morphology, T2‐weighted and diffusion‐weighted imaging features, enhancement patterns, and associated findings; apparent diffusion coefficient values were measured.

**Results:**

All ICTNs were lobulated, nearly spherical intraluminal masses with a thin stalk (17.5 ± 2.7 mm). On T2‐weighted MRI, lesions showed predominantly low signal intensity with fissure‐like or punctate hyperintense foci. Diffusion‐weighted imaging showed hyperintensity with mildly reduced apparent diffusion coefficient values (1.77 ± 0.27 × 10^−3^ mm^2^/s). Dynamic imaging revealed avid arterial enhancement with gradual washout or a plateau pattern in the venous phase. Histologically, compact tubular glands were separated by narrow, vascular‐rich septa, corresponding to the imaging findings.

**Conclusion:**

Our findings suggest that a combination of a pedunculated, “raspberry‐like” mass, arterial enhancement with mild late‐phase washout on dynamic CT, and diffusion restriction on MRI may provide useful imaging clues for differentiating ICTN from gallbladder malignancies.

## 1. Introduction

The most common tumorous lesions of the gallbladder include carcinoma and cholesterol polyps, whereas preinvasive lesions are infrequently encountered. In the 6th edition of the World Health Organization (WHO) Classification of Tumours of Digestive System, mass‐forming intraepithelial neoplasms of the gallbladder are provisionally designated as intracholecystic neoplasms (ICNs) and are further subcategorized into intracholecystic papillary neoplasm (ICPN) and intracholecystic tubular neoplasm (ICTN) [1]. ICTN, originally described as “intracholecystic tubular nonmucinous neoplasm,” is a newly recognized entity characterized by a mass‐forming lesion arising within a gallbladder lumen that resembles ICPN grossly but has distinct histopathologic features [2–4]. Despite increasing recognition of ICTN as a distinct pathological entity, its imaging features and radiologic–pathologic correlation have not been well described. To our knowledge, this study represents the first radiologic–pathologic case series of ICTN.

## 2. Materials and Methods

### 2.1. Patients

This retrospective study was approved by the institutional review boards of two participating institutions. Between March 2021 and June 2025, four patients underwent cholecystectomy at our institutions, and the resected specimens were pathologically diagnosed as ICTN. All lesions were initially detected on screening ultrasonography or imaging performed for other conditions, and surgical resection was indicated because the polyps exceeded 10 mm in size, raising suspicion for malignancy.

### 2.2. Pathological and Imaging Analysis

A histological diagnosis was made independently by two pathologists at each institution according to the criteria of Pehlivanoglu et al. [2]. Routine hematoxylin–eosin staining and immunohistochemistry were performed.

Preoperative imaging studies included ultrasonography, contrast‐enhanced computed tomography (CT), and magnetic resonance imaging (MRI). Two board‐certified radiologists (with 12 and 10 years of diagnostic radiology experience) independently reviewed the images, and discrepancies were resolved by a consensus. Furthermore, all radiologists among the authors reviewed the extracted imaging findings. Imaging examinations were performed according to the respective protocols of the two institutions.

Dynamic CT studies for all patients evaluated noncontrast, arterial phase (late arterial phase), portal venous phase, and venous phase with a slice thickness of 5 mm using an iodinated contrast agent. MRI was performed using an Ingenia 3.0T (Philips, Amsterdam, The Netherlands) scanner at both institutions. The MRI protocol included T2‐weighted images (using half Fourier single‐shot turbo spin echo), magnetic resonance cholangiopancreatography, diffusion‐weighted images (*b* = 800 s/mm^2^), and apparent diffusion coefficient (ADC) mapping. To perform dynamic contrast‐enhanced MRI studies in Cases 1 and 3, fat‐suppressed T1‐weighted images were obtained at the precontrast, arterial, portal venous, and venous phases. Gadovist (gadobutrol), an extracellular gadolinium‐based contrast agent was administered for all contrast‐enhanced MRI sequences. The slice thickness for Cases 1, 3, and 4 were 3 mm for T2‐weighted imaging (T2WI), 4 mm for T1WI, 5 mm for diffusion‐weighted imaging (DWI), and 2.6 mm for magnetic resonance cholangiopancreatography. In Case 2, the slice thicknesses were 5 mm for T2WI and DWI, 6 mm for T1WI, and 1.6 mm for magnetic resonance cholangiopancreatography.

The tumor size was measured as the maximal diameter on the most recent preoperative imaging. ADC values were measured by placing a region of interest over the largest cross‐sectional area of the tumor, and the mean value was recorded.

### 2.3. Statistical Analysis

Data are expressed as the mean ± standard deviation. Statistical analyses were performed using EZR software (Saitama Medical Center, Jichi Medical University, Saitama, Japan) [5], which is a graphical user interface for *R* (The *R* Foundation for Statistical Computing, Vienna, Austria). Only studies of diagnostic quality were included in the analysis.

## 3. Results

### 3.1. Clinical Features

All four patients were asymptomatic, and their gallbladder masses were incidentally detected on ultrasonography or CT. The patients ranged in age from 49 to 71 years (mean: 64.3 ± 10.3 years), and all of them had normal preoperative CEA and CA 19–9 concentrations.

### 3.2. Imaging Findings

All cases showed similar imaging features. Representative images are shown in Figures 1, 2, 3, and 4 and summarized in Table 1. The tumors appeared as pedunculated intraluminal masses with a lobulated, nearly spherical configuration. They measured 15–21 mm (mean: 17.5 ± 2.7 mm) and were attached by a thin stalk measuring 1.1–1.5 mm in diameter. On ultrasonography, the lesions appeared as mildly hyperechoic polypoid lesions, while the thin stalk was difficult to visualize. On plain CT, the lesions showed mild hyperattenuation. On dynamic contrast‐enhanced CT, Cases 1, 2, and 4 showed arterial phase enhancement followed by washout, whereas Case 3 showed mild arterial phase enhancement followed by a plateau pattern. On MRI, the masses showed a predominantly low signal on T2WI with fissure‐like or punctate hyperintense foci. DWI showed hyperintensity with mildly reduced ADC values (1.77 ± 0.27 × 10^−3^ mm^2^/s). Dynamic contrast‐enhanced MRI was performed in Cases 1 and 3; Case 1 showed inconspicuous early enhancement with delayed enhancement, whereas Case 3 showed early enhancement with a plateau pattern. No associated gallbladder distension, gallstones, or bile duct dilatation was observed in any case.

**FIGURE 1:**
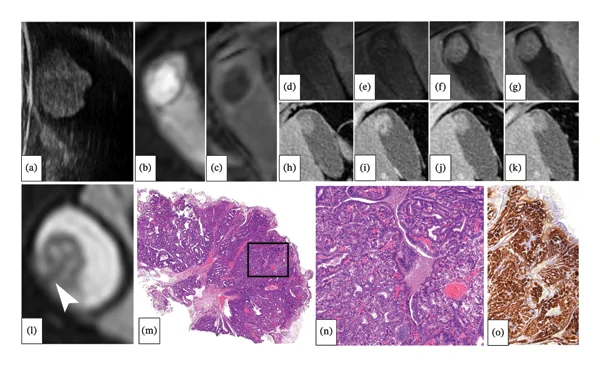
Case 1. (a) A gallbladder polypoid mass was detected on ultrasonography during a routine health checkup. (b) MRI diffusion‐weighted image (*b* = 800). The mass shows high signal intensity. (c) MRI ADC map. The mass shows low values. (d–g) Dynamic contrast‐enhanced MRI. (d) Precontrast fat‐suppressed T1‐weighted image, (e) arterial phase, (f) portal venous phase, and (g) venous phase. The mass shows delayed enhancement. (h–k) Dynamic contrast‐enhanced CT. (h) Precontrast CT, (i) arterial phase, (j) portal venous phase, and (k) venous phase. The lesion shows arterial enhancement with mild washout. (l) MRI T2‐weighted image. A spherical mass with a thin stalk (white arrowhead) is present within the gallbladder, showing overall low signal intensity with a cracked‐like area of high signal intensity. (m) Hematoxylin–eosin (HE)–stained section in a magnified view. The mass is lobulated and detached from the gallbladder wall. (n) Magnified view of the area outlined by the black box in. The atypical cells formed tubular structures. (o) MUC6 staining is diffusely positive.

**FIGURE 2:**
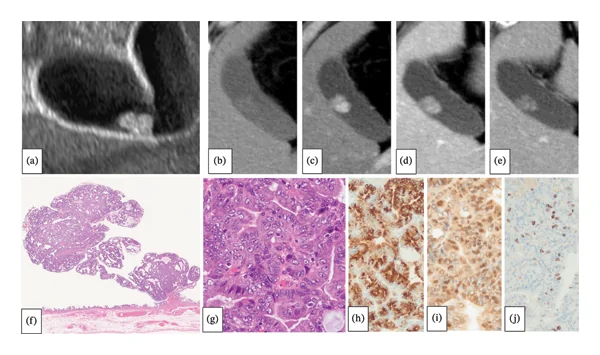
Case 2. (a) A hyperechoic mass attached to the gallbladder wall was incidentally found on ultrasonography and slightly enlarged over 2 years. (b–e) Dynamic contrast‐enhanced CT. (b) Precontrast CT, (c) arterial phase, (d) portal venous phase, and (e) venous phase. The lesion shows strong enhancement in the arterial phase and washout in the venous phase. (f) Hematoxylin–eosin (HE)–stained section in a magnified view. The lobulated mass is attached to the gallbladder wall mucosa by a thin stalk‐like structure. (g) HE‐stained section at high magnification. The atypical cells formed tubular structures. (h) MUC6 staining is diffusely positive. (i) *β*‐catenin staining is diffusely positive. (j) CD56 staining is positive.

**FIGURE 3:**
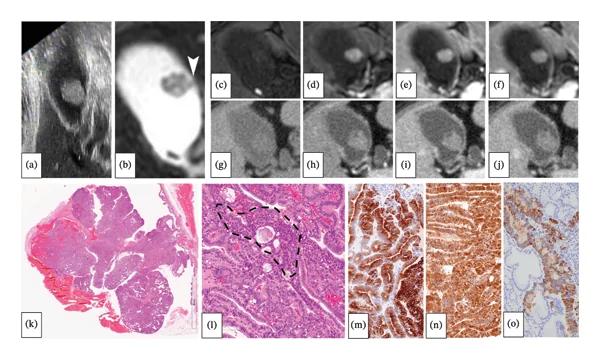
Case 3. (a) Ultrasonography demonstrated a slowly growing hyperechoic gallbladder mass over 2 years. (b) MRI T2‐weighted image. A mildly lobulated mass with a thin stalk (white arrowhead) is present within the gallbladder, showing overall low signal intensity with punctate areas of high signal intensity. (c–f) Dynamic contrast‐enhanced MRI. (c) Precontrast fat‐suppressed T1‐weighted image, (d) arterial phase, (e) portal venous phase, and (f) venous phase. The mass shows early arterial enhancement with persistent delayed enhancement through the venous phase. (g–j) Dynamic contrast‐enhanced CT. (g) Precontrast CT, (h) arterial phase, (i) portal venous phase, and (j) venous phase. The mass shows mild arterial enhancement with a plateau pattern. (k) Hematoxylin–eosin (HE)–stained section in a magnified view. The lobulated mass is attached to the gallbladder wall mucosa by a thin stalk‐like structure. (l) A high magnification view shows morule formation (within the black dashed area) (HE stain). (m) MUC6 staining is diffusely positive. (n) *β*‐catenin staining shows positive nuclear expression. (o) Synaptophysin is focally positive.

**FIGURE 4:**
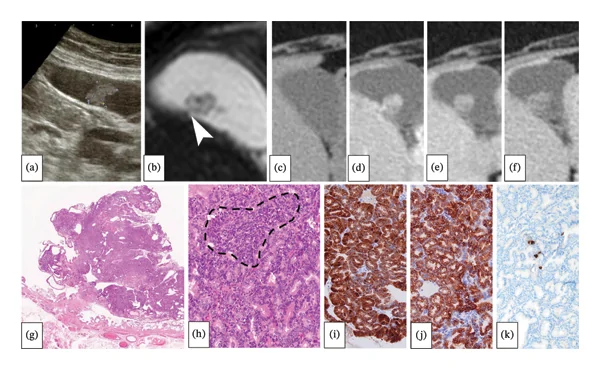
Case 4. (a) Ultrasonography incidentally showed a lobulated, hyperechoic mass attached to the gallbladder wall. (b) MRI T2‐weighted image shows a mildly lobulated mass attached to the gallbladder wall by a thin stalk, showing overall low signal intensity with punctate areas of high signal intensity. (c–f) Dynamic contrast‐enhanced CT. (c) Precontrast CT, (d) arterial phase, (e) portal venous phase, and (f) venous phase. The mass shows arterial hyperenhancement and washout in the venous phase. (g) Hematoxylin–eosin (HE)–stained section in a magnified view. The lobulated mass is attached to the gallbladder wall mucosa by a thin stalk‐like structure. (h) A high magnification view shows morule formation (within the black dashed area) (HE stain). (i) MUC6 staining is diffusely positive. (j) *β*‐catenin staining shows positive nuclear expression. (k) Chromogranin A staining is partially positive.

**TABLE 1:** Summary of cases and imaging findings.

	Age	Sex	Size (mm)	Form	SI on T2WI	SI on DWI	ADC value (× 10^–3^ mm^2^/sec)	Dynamic CT	Dynamic MRI
Case 1	49	M	18 × 15 × 11	Pedunculated	Low with high tiny foci	High	1.43	Arterial hyperenhancement, mild washout	Delayed enhancement
Case 2	67	M	21 × 14 × 8	Pedunculated	Low	High	1.86	Arterial hyperenhancement, delayed washout	—
Case 3	71	F	16 × 14 × 11	Pedunculated	Low with high tiny foci	High	1.72	Arterial enhancement, plateau	Arterial enhancement, plateau
Case 4	70	F	15 × 10 × 9	Pedunculated	Low with high tiny foci	High	2.08	Arterial hyper enhancement, delayed washout	—

Abbreviations: ADC, apparent diffusion coefficient; DWI, diffusion‐weighted imaging; SI, signal intensity; T2WI, T2‐weighted imaging.

### 3.3. Pathological Findings

Pathological images for each case (Figures 1, 2, 3, and 4) showed a fragile, lobulated morphology under a loupe examination. Histologically, the tumors consisted of cylindrical atypical epithelium proliferating in tubular and papillary patterns with fused glandular ducts. In Cases 2–4, residual stalks with retracted mucosa were identified. Nuclear atypia corresponding to high‐grade dysplasia was observed in all cases, and no evidence of invasion was identified. In Cases 2–4, squamoid/meningothelial‐like morules were observed. In Case 2, clusters of foamy cells were sporadically observed beneath the superficial epithelium near the base and apex of the tumor. Only Case 3 showed a small amount of luminal mucin without intracellular mucin. Immunohistochemically, all cases showed diffuse MUC6 positivity. MUC5AC staining was partially positive or negative in Cases 1–3. Chromogranin A or synaptophysin showed focal positivity in certain areas. In all cases, p53 showed a wild‐type pattern, and partial nuclear expression of *β*‐catenin was observed in Cases 2–4.

## 4. Discussion

### 4.1. Imaging–Pathological Correlation

Histologically, ICTN is characterized by a pedunculated polyp with a thin stalk, tightly packed small acinar‐like tubular units, minimally mucinous or nonmucinous epithelium, and high‐grade dysplasia without invasion. Immunohistochemically, ICTN typically shows diffuse MUC6 positivity, rare MUC5AC expression, morule formation, and nuclear accumulation of *β*‐catenin [2, 4]. However, immunohistochemistry serves primarily as ancillary support, and no specific immunohistochemical markers or staining profiles have been established as diagnostic requirements for ICTN [2, 4]. The histological findings in all four of our cases were compatible with the characteristic morphological features of ICTN. On the basis of these histopathological characteristics, we evaluated the radiological features of our four cases in relation to the pathological findings. On CT and MRI, ICTN typically appeared as a nodular, lobulated mass, frequently with a thin stalk. Although a thin stalk was pathologically confirmed in all cases except Case 1, where it was likely fragmented during surgical resection, it was radiologically identified only on 3‐mm T2WI in Cases 1, 3, and 4 and not on 5‐mm slices in Case 2, highlighting the critical role of thin‐section T2WI for detection of the thin stalk. On MRI, the lesions appeared as lobulated intraluminal tumors, characterized by fissure‐like hyperintense areas within a predominantly low T2 signal. These imaging features reflected the underlying histology of compact tubular structures interspersed with occasional microcystic or hyalinized areas. While ultrasonography clearly showed overall spherical morphology, the internal architecture showed nonspecific hyperechogenicity.

Dynamic CT demonstrated arterial‐phase enhancement followed by washout in three cases, whereas one case showed mild arterial‐phase enhancement followed by a plateau pattern. On dynamic MRI, Case 1 showed inconspicuous early enhancement, likely due to premature acquisition before the true arterial phase, whereas Case 3 showed early enhancement with a plateau pattern. The early hypervascularity may reflect the rich vascularity of the tumor base, while the stromal component associated with the ductal architecture may contribute to prolonged delayed enhancement.

Preoperative imaging showed no evidence of cholecystitis, cholelithiasis, or features suggestive of tumor invasion, which was consistent with the pathological findings. Although the cytoarchitectural atypia corresponded to high‐grade dysplasia, there was no histological evidence of invasion into the gallbladder wall.

### 4.2. Differential Diagnosis

Differential diagnoses for pedunculated gallbladder masses include benign cholesterol polyps, preinvasive ICPN, and malignant mass‐forming carcinoma. A cholesterol polyp is the most common polypoid lesion found in the gallbladder and is a typically small (< 1 cm), multiple, and round intraluminal lesion attached to the wall, and the stalk is rarely observed [6]. ICTN appears to arise in cholesterol polyps; 32% of ICTNs have cholesterolosis within the polypoid lesion pathologically [2]. ICPN is a rare, macroscopic gallbladder precursor that frequently presents without wall abnormalities and is characterized by broad‐based or papillary masses and mucin hypersecretion [7, 8]. These low‐ or high‐grade lesions progress to invasive carcinoma in 26.9%–55% of cases [3, 9]. Adenocarcinoma of the gallbladder is the most common malignant gallbladder polyp. In 15%–25% of cases, adenocarcinoma manifests as an intraluminal polyp with a wide polyp vase, focal wall thickening, and coexisting gallstones [6].

Morphologically, ICTN closely resembles these differential diagnoses, making it challenging to distinguish from other polypoid gallbladder masses. Notably, a thin stalk was identified on MRI in all of our cases, with none presenting as sessile. This characteristic feature may provide a critical diagnostic clue for ICTN. Therefore, we propose the term “raspberry‐like appearance” to describe ICTN, characterized by a pedunculated, lobulated gallbladder mass with a slender stalk measuring approximately 1–2 mm in diameter. Importantly, as shown in Cases 1, 3, and 4, this thin stalk could be identified on careful multiplanar review of 3‐mm‐thick T2WI, including coronal and sagittal views. Therefore, identification of this thin stalk on MRI may serve as a useful radiological sign of differentiating ICTN, particularly from conventional gallbladder adenocarcinoma, which typically presents as a sessile lesion with broad‐based attachment to or replacement of the gallbladder wall.

We also evaluated the dynamic contrast enhancement patterns and ADC values on MRI. On dynamic MRI, cholesterol polyps typically show arterial hyperenhancement followed by washout in the venous phase [10]. In contrast, gallbladder cancer and ICPN have been reported to show arterial hyperenhancement with prolonged enhancement in the venous phase [11]. Our ICTN cases showed arterial hyperenhancement followed by mild washout on dynamic CT, representing an intermediate enhancement pattern between cholesterol polyps and gallbladder cancer or ICPN.

Diffusion restriction on MRI is useful for differentiating benign from malignant gallbladder lesions. Reported ADC values range from 0.97 to 1.83 × 10^−3^ mm^2^/s for gallbladder cancer and from 1.72 to 2.6 × 10^−3^ mm^2^/s for benign lesions [7, 12–15]. The mean ADC value of our ICTN cases (1.77 × 10^−3^ mm^2^/s) fell within the overlapping zone of these two ranges, indicating moderate diffusion restriction. This intermediate value is pathologically consistent with the dense tubular architecture of ICTN and may aid in distinguishing it from gallbladder carcinoma and cholesterol polyps. A previous case report of ICPN showed an ADC value of 1.52 × 10^−3^ mm^2^/s [11], suggesting diffusion restriction comparable to or slightly stronger than that observed in ICTN.

### 4.3. Clinical Implications and Management

ICTN is a recently recognized, distinct mass‐forming gallbladder lesion characterized by its invasion‐resistant behavior and remarkably favorable prognosis. The principal clinical importance of accurately identifying ICTN lies in avoiding overtreatment. In contrast to ICPNs, which have a substantial risk of progression to conventional invasive gallbladder carcinoma, ICTNs are effectively treated by simple cholecystectomy alone. When complete margin‐negative resection is achieved, additional radical procedures, including extended cholecystectomy and lymph node dissection, are generally unnecessary. Therefore, distinguishing ICTN from more aggressive gallbladder neoplasms is essential not only to prevent unnecessary surgical intervention but also to provide appropriate prognostic information. Recognition of the characteristic features of ICTN may facilitate its differentiation from other gallbladder polypoid lesions and guide optimal surgical management while minimizing procedure‐related morbidity.

### 4.4. Limitations

This study has several limitations. First, the sample size was small because ICTN is extremely rare and has only recently been proposed as a distinct entity; therefore, further accumulation of cases is needed. Second, this was a retrospective review from two institutions, and the imaging equipment and protocols were not standardized. To minimize this bias, radiologists from both institutions jointly reviewed all imaging findings. Third, regarding radiopathological correlation, the thin stalk identified on MRI in Case 1 could not be confirmed in the surgical specimen, likely due to detachment or fragmentation during resection. Finally, immunohistochemical evaluation was not uniform across all cases, as definitive diagnostic markers for ICTN have not yet been fully established.

## 5. Conclusion

To our knowledge, this is the first case series focusing on the imaging characteristics of ICTN. Although limited by the small number of cases, our findings suggest that a combination of imaging features—including a “raspberry‐like” appearance, a pedunculated configuration with a thin stalk, arterial enhancement with mild late‐phase washout or a plateau pattern on dynamic CT, and moderate diffusion restriction on MRI—may provide useful imaging clues for differentiating ICTN from other gallbladder malignancies and may contribute to preoperative diagnosis and management planning.

## Funding

This work was supported by Grant for Assist KAKEN from Kanazawa Medical University (K2026‐22).

## Conflicts of Interest

The authors declare no conflicts of interest.

## Data Availability

The data that support the findings of this study are not openly available due to reasons of sensitivity.
